# Supplementary material for: The Effect of Elasticity of Gelatin Nanoparticles on the Interaction with Macrophages
Source: Pharmaceutics. 2023 Jan 6;15(1):199. doi: 10.3390/pharmaceutics15010199 (PMC9861130; doi:10.3390/pharmaceutics15010199)
Supplement: Supplementary file 1 [file pharmaceutics-15-00199-s001.zip › pharmaceutics-2082294-supplementary.pdf]

Supplementary Material

The Effect of Elasticity of Gelatin Nanoparticles on the Interaction with Macrophages

Metin Yildirim [1,2], Agnes-Valencia Weiss [1] and Marc Schneider [1,*]

[1] Department of Pharmacy, Biopharmaceutics and Pharmaceutical Technology, Saarland University, 66123 Saarbrücken, Germany
[2] Department of Pharmacy Services, Vocational School of Health Services, Tarsus University, 33400 Mersin, Turkey
* Correspondence: marc.schneider@uni-saarland.de

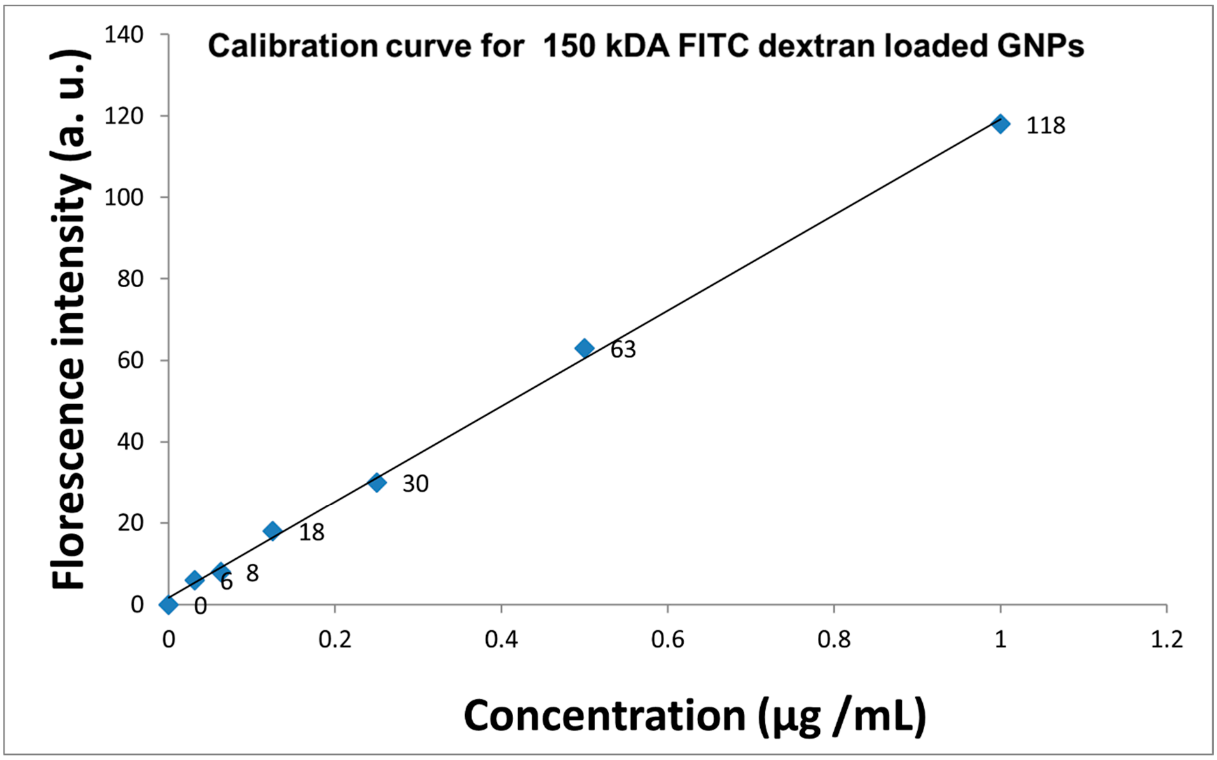

Figure S1: Calibration curve of 150 kDA FITC-dextran-loaded GNPs

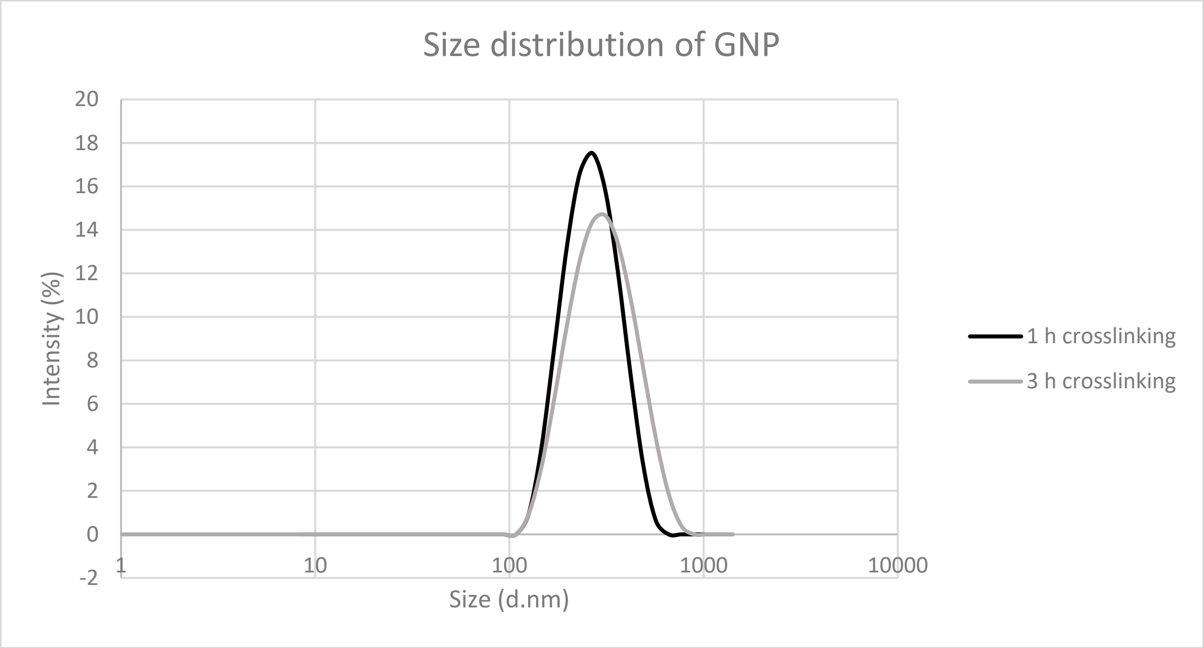

Figure S2: Size distribution of GNPs crosslinked for 1 and 3 h. Data represents a representative measurement for each formulation
